# Sushi hand roll dressing for thumb polydactyly

**DOI:** 10.1186/1749-799X-7-26

**Published:** 2012-06-12

**Authors:** Chi-Hung Yen, Pak-Cheong Ho, Leung-Kim Hung

**Affiliations:** 1 Department of Orthopaedics and Traumatology, Kwong Wah Hospital, The Hong Kong Special Administrative Region, Kowloon, Hong Kong; 2 Department of Orthopaedics and Traumatology, Prince of Wales Hospital, The Hong Kong Special Administrative Region, Kowloon, Hong Kong

**Keywords:** Polydactyly, Wound, Dressing

## Abstract

Surgery for thumb polydactyly is a commonly performed orthopaedic procedure in Asia Pacific region. Despite extensive publications on topical dressing methods and dressing materials in paediatric wounds, there is no single design that affords a secure and yet comfortable post-operative wound dressing for thumb polydactyly. We have devised a new dressing method, which can easily be fabricated for such purpose from readily available materials in operation theatre.

## Background

Thumb polydactyly is the most common congenital anomaly in upper limb in Asia Pacific region [1,2]. The incidence of thumb polydacyly constitutes approximately less than 10% of upper-extremity congenital abnormalities [1]. Surgery for thumb polydactyly is therefore a commonly performed orthopedic procedure, which is usually performed after 1 year of age weighing between risks of general anesthesia and benefits of early cortical re-education [3]. As the child is not mature enough to abide by the dressing care of surgical wound in which he is obsessive to remove anything that covers the hand, it is of paramount importance to ensure a secure and yet comfortable post-operative wound dressing for thumb polydactyly. Despite extensive publications on topical dressing methods and dressing materials in pediatric wounds, there is no single design of wound dressing to cater for the above needs [4-11]. We have devised a new dressing method, which can easily be fabricated for such purpose from readily available materials in operation theatre.

## Methods

**Figure 1:**
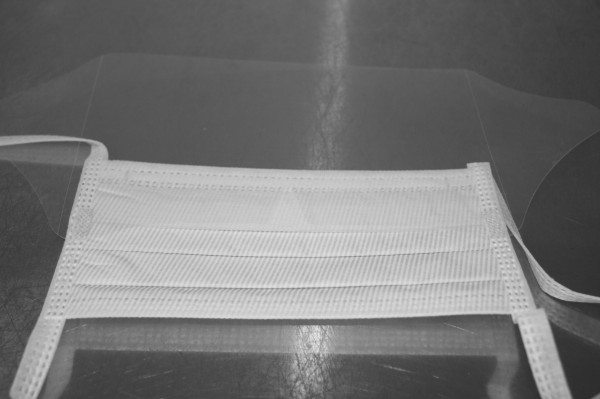
The transparent face shield is detached from the mask by peeling it off from its attachments to the mask.

**Figure 2:**
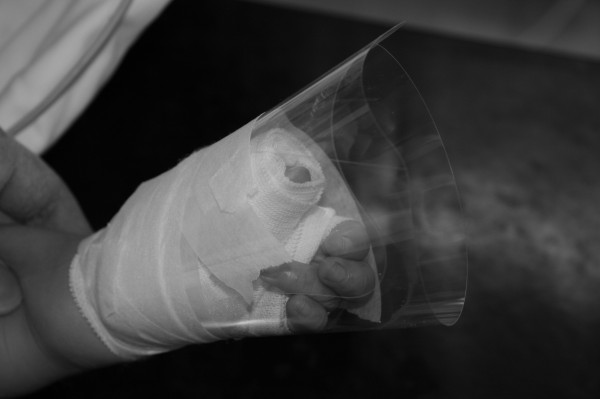
**It is then rolled over the ordinary bandage dressing as sushi hand roll, securely held in place at wrist level with 1-inch wide 3M**^**TM**^**Micropore**^**TM**^**medical tape.**

**Figure 3:**
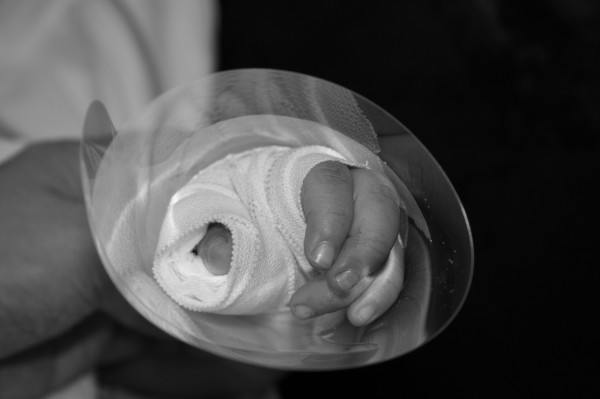
It is imperative to expose tips of all fingers for observation of circulation and most importantly the additional benefit of reassurance to the child that all fingers are still visualized and movable by him.

The only material required is the transparent face shield of a surgical mask. The transparent face shield is detached from the mask by peeling it off from its attachments to the mask (Figure 1). It is then rolled over the ordinary bandage dressing in a sushi hand roll manner, securely held in place at wrist level with 1-inch wide 3M^TM^ Micropore^TM^ medical tape (1530-1) (Figure 2). In this connection, additional taping is not necessary to be put on the original dressing. It is imperative to expose tips of all fingers for observation of circulation and most importantly the additional benefit of reassurance to the child that all fingers are still visualized and movable by the child (Figure 3).

## Results and discussion

Despite simplicity of this dressing method, the physical exposure and visibility of all fingers in the dressing are the keys to success of this dressing method. A child will not attempt to remove the dressing because of the reasons stated therein and yet he cannot remove bandage through the transparent face shield. Otherwise, the child will keep on removing whatever dressing which obscures the fingers as the patient used to see and move fingers beforehand. On the one hand, many dressing methods for different paediatric conditions involving tracheostomy, eye and penis have shown promising results [7-10]. In spite of numerous recent reports on thumb polydactyly, few studies have mentioned a secure and yet comfortable post-operative dressing method [1-3,12,13]. Furthermore, the child may think the sushi hand roll dressing as a new toy and would like to play with it rather than removing it. This new method translates Elizabethan collar into cone of fun for the child. The sushi hand roll dressing can be kept as long as 2 weeks by which non-absorbable sutures will be removed. Edge of the plastic sheet is of prime concern regarding safety to the child. The edge by itself is smooth, soft and malleable to touch and pressure. In addition to meeting European standards of EN14683.2005 Type IIR, CE marking on Molnlycke Barrier^@^ extra protection visor face mask REF4232 is a manufacturer’s declaration that the product compiles with the essential requirements of the relevant European health, safety and environmental protection legislation. Since 2003 in our department, the sushi hand roll dressing is the standard protocol of post-operative wound dressing for our 39 patients with thumb polydactyly. It is authors’ experience that no known or reported personal injury has been incurred as a result of the plastic shield. We have also successfully extended the indication to other pediatric problems including syndactyly, hypoplastic thumb, congenital trigger thumb, and burns. Because of the timing of surgery in early childhood, the child cannot remember the event of an extra digit when the patient is asked at latest follow-up during adolescence [14]. There is no reported case of bandage removal by the child.

## Conclusions

A new post-operative dressing method has been devised for paediatric patients after surgery for thumb polydactyly. It not only provides a safe, but also securely comfortable dressing. The indication of sushi hand roll dressing can be extended to other pediatric hand conditions like syndactyly, hypoplastic thumb, congenital trigger thumb, and burns.

## Competing interests

The authors declare that they have no competing interests.

## Authors’ contributions

LKH conceived the idea. PCH and CHY performed the surgery. LKH and PCH contributed to the discussions. All authors have read and approved the final manuscript.
